# Modulation of Cell Response through the Covalent Binding of Fibronectin to Titanium Substrates

**DOI:** 10.3390/jfb14070342

**Published:** 2023-06-28

**Authors:** Parsa Rezvanian, Aroa Álvarez-López, Raquel Tabraue-Rubio, Rafael Daza, Luis Colchero, Manuel Elices, Gustavo V. Guinea, Daniel González-Nieto, José Pérez-Rigueiro

**Affiliations:** 1 Center for Biomedical Technology, Universidad Politécnica de Madrid, Pozuelo de Alarcón, 28223 Madrid, Spain; parsa.rezvanian@gmail.com (P.R.); aroa.alvarez@ctb.upm.es (A.Á.-L.); rafael.daza@upm.es (R.D.); gustavovictor.guinea@ctb.upm.es (G.V.G.); daniel.gonzalez@ctb.upm.es (D.G.-N.); 2 Departamento de Ciencia de Materiales, ETSI Caminos, Canales y Puertos, Universidad Politécnica de Madrid, 28040 Madrid, Spain; m.elices@upm.es; 3 Department of Animal Biotechnology, Cell Science Research Center, Royan Institute for Biotechnology, ACECR, Isfahan 8159358686, Iran; 4 Bioactive Surfaces S.L. C/Puerto de Navacerrada 18, Galapagar, 28260 Madrid, Spain; raquel.tabraue@ctb.upm.es (R.T.-R.); luis.colchero@ctb.upm.es (L.C.); 5 Biomedical Research Networking Center in Bioengineering, Biomaterials and Nanomedicine (CIBER-BBN), 28029 Madrid, Spain; 6 Instituto de Investigación Sanitaria del Hospital Clínico San Carlos (IdISSC), Calle Prof. Martín Lagos s/n, 28040 Madrid, Spain; 7 Departamento de Tecnología Fotónica y Bioingeniería, ETSI Telecomunicaciones, Universidad Politécnica de Madrid, 28040 Madrid, Spain

**Keywords:** fibronectin, functionalization, activated vapor silanization (AVS), mesenchymal stem cells (MSC), biomaterial

## Abstract

Titanium (Ti-6Al-4V) substrates were functionalized through the covalent binding of fibronectin, and the effect of the existence of this extracellular matrix protein on the surface of the material was assessed by employing mesenchymal stem cell (MSC) cultures. The functionalization process comprised the usage of the activation vapor silanization (AVS) technique to deposit a thin film with a high surface density of amine groups on the material, followed by the covalent binding of fibronectin to the amine groups using the N-(3-dimethylaminopropyl)-N′-ethylcarbodiimide hydrochloride/N-hydroxysuccinimide (EDC/NHS) crosslinking chemistry. The biological effect of the fibronectin on murine MSCs was assessed in vitro. It was found that functionalized samples not only showed enhanced initial cell adhesion compared with bare titanium, but also a three-fold increase in the cell area, reaching values comparable to those found on the polystyrene controls. These results provide compelling evidence of the potential to modulate the response of the organism to an implant through the covalent binding of extracellular matrix proteins on the prosthesis.

## 1. Introduction

In recent decades, there has been a progressive demand for biomaterials with the capacity to serve as substitutes for a wide range of tissues and replicate their functions [1,2]. Titanium (Ti) has been the material of choice for many of these applications owing to its convenient combination of properties: excellent corrosion resistance, relatively low elastic modulus, and high tensile strength [3,4]. Given these remarkable attributes, titanium biomaterials have emerged as viable candidates for applications as implants and replacement of hard tissue. However, Ti, as is the case for most if not all of the biomaterials used in clinical practice, exhibits a major drawback related to the interaction established with the surrounding living tissue: upon implantation the material lacks the ability to establish intimate direct contact with the surrounding tissues and is covered by a connective tissue capsule. The formation of this connective tissue capsule is an inherent consequence of the foreign body reaction elicited by the recipient organism towards the implant. The foreign body reaction implies the activation of the macrophages and, together with active fibroblasts, the generation of a collagen matrix around the implant [5,6].

The absence of proper integration between an implant and the surrounding tissue can be problematic, since a weak connection between the implant and healthy tissue may give rise to infections [7]. Furthermore, the presence of a fibrous tissue interface, which is mechanically weak in nature, causes micro-movements of the implant relative to the adjacent tissue. These micro-movements can detrimentally impact the healing process and ultimately result in the loosening of the implant over time [8,9]. Implant loosening, in turn, may cause pain and often necessitates revision surgery. For instance, it is reported that the loosening of the implant is the primary cause for revision hip arthroplasty [10].

To address this issue and to promote the integration of the material, it is imperative to actively attract stem cells to the surface of the implant and to promote their biological activity. Such an approach holds the potential for the direct formation of the functional tissue directly on the surface of the prosthesis. Alternatively, controlling the cell/material interaction offers the possibility of culturing and differentiating cell lineages in vitro prior to implantation in the patient, so that this biological layer, and not the material itself, will be in contact with the surrounding tissue.

Incidentally, a strategy involving surface modification of implants with biomolecules seems adequate and leads to the so-called biofunctionalization of the material. Biofunctionalization consists of modifying the surface of the material, usually through the binding of targeted biomolecules, in an attempt to promote an enhanced biological response of the organism to the implant. It is assumed that this enhanced response is greatly dependent on the existence of specific adhesion sites for different cells at the surface of the material [11]. Although it is possible to immobilize the biomolecules through such simple processes as adsorption or entrapment, the creation of a robust connection between the material and the biomolecule usually requires the formation of a covalent bond, which implies the generation of functional groups on the surface of the implant. For instance, in the case of titanium, the conventional approach to functionalization involves submerging the substrate in a solution of an organosilane in an organic solvent, such as pentane or toluene [12,13,14,15]. Although this method is simple and facile, it lacks reproducibility and leads to the formation of a relatively low surface concentration of functional groups. This is due to the high susceptibility of the silanization reactions to hydrolyzation and oligomerization of the precursor molecules [16,17]. Thus, a functionalization method overcoming these disadvantages may greatly improve the biomolecule binding efficacy.

In this context, the usage of the AVS process allows creating a stable and robust covalent bond between the surface of the material and any biomolecule of interest, and overcomes the reproducibility issues typically associated with other silanization procedures. This way, and taking the AVS procedure as an adequate starting point, it is necessary to select the biomolecule to be immobilized, as well as the cells whose interaction with the surface is to be modulated.

Following this rationale, fibronectin appears as a specially interesting candidate as a biologically active molecule with which the titanium substrate may be decorated in order to control the response of the cells and prevent spurious reactions that might lead to the failure of the prosthesis. This choice is supported by the significant role that fibronectin plays in the interaction between cells and the extracellular matrix (ECM).

Fibronectin possesses binding sites for heparin, fibrin, and collagen [18], as well as several integrin binding sites which may be recognized by different cell lines [19,20,21,22]. Fibronectin is found to favor cell adhesion [23,24] and spreading [25,26]. Hence, coating the surface of biomaterials with fibronectin has been a viable strategy for enhancing cellular response to the biomaterials. The positive effect of fibronectin adsorbed on the surface of Ti biomaterials on the cellular response has been addressed previously in various studies. It was reported that adsorption of fibronectin on Ti-6Al-4V alloy from a solution of the protein with a concentration as low as 0.5 nM can lead to a substantial improvement in the cell attachment to the material [27]. Previously, improved MC3T3-E1 cell adhesion and proliferation on fibronectin-coated Ti was reported by Ku et al. [28]. Adsorption of fibronectin on Ti materials has been demonstrated to improve adhesion and proliferation of human gingival fibroblasts [29], hamster kidney 21C/13 fibroblasts [30], MG63 osteoblasts, C3H10T1/2 mesenchymal stem cells [27], rat bone marrow-derived osteoblasts [31], and normal human dermal fibroblasts [32].

In addition to these non-covalent strategies, several procedures for the covalent binding of fibronectin to solid substrates have been also developed. Thus, fibronectin was immobilized on Ti alloys using the tresyl chloride chemistry, demonstrating a positive impact on the adhesion of MC3T3-E1 cells [33,34]. In a similar study, Pham et al. functionalized Ti samples using immersion silanization and EDC as crosslinker, and concluded that at 4 h after seeding, osteoblast-like SaOS2 cells exhibited enhanced cell adhesion and spreading on fibronectin-decorated samples [35]. These functionalization procedures, however, tend to be heavily reliant on the detailed surface chemistry and, consequently, lead to a high variability in the observed outcomes.

With regard to the cell lineage, bone marrow mesenchymal stem cells (BM-MSCs) represent a convenient choice, due to their ability to differentiate into osteoblasts and, consequently, to produce bone tissue. The differentiation of BM-MSCs on the surface of the material is a promising strategy for preventing the formation of the connective tissue capsule around the implant.

In this study, we show how it is possible to covalently bind fibronectin to the surface of biomaterials using a functionalization procedure that is largely independent of the chemistry of the substrate. Ti-6Al-4V is used as a model system and functionalized through the activated vapor silanization (AVS) process, resulting in a high density of reactive amines on the surface of the material. Subsequently, fibronectin is bound to the functionalized titanium surface through the EDC/NHS crosslinking chemistry. Lastly, the enhanced response of murine bone marrow mesenchymal stem cells (BM-MSCs) on the fibronectin-decorated titanium samples is verified.

## 2. Materials and Methods

### 2.1. Preparation of Ti Substrates

Substrates used in this study were cut from an ingot of commercial Ti-6Al-4V alloy with nominal dimensions of 10 × 10 × 1 mm. The substrates were polished sequentially with sandpapers having grit No. 80, 400, 1200, and 4000. Subsequently, the samples underwent a thorough cleaning process using sonication in acetone, isopropanol, and distilled water, and dried with a flow of argon.

### 2.2. Covalent Immobilization of Fibronectin

#### 2.2.1. Functionalization

The surfaces of the Ti substrates were initially amino-functionalized using the activated vapor silanization (AVS) method, following a previously described protocol [36,37]. Briefly, 3-aminopropyltriethoxysilane (APTS, Fluka, Madrid, Spain) is poured into a sealed compartment and evaporated at low vacuum. The resulting vapor is transported by an argon flux (BIP, Purity ≥ 99, 9997%) to an activation chamber where the temperature is elevated to 750 °C. Afterwards, the activated APTS vapor is directed towards the surface of the substrates within the deposition chamber. Finally, the vapor phase is evacuated from the system using a rotary pump. The process is controlled by four parameters: evaporation temperature of APTS (T_Evap_), activation temperature of APTS (T_Act_), pressure of argon (P_Ar_), and deposition time (*t*). For the functionalization of Ti substrates in this study, the deposition parameters were set as follows: T_Evap_ = 150 °C, T_Act_ = 750 °C, P_Ar_ = 2 mbar, and *t* = 20 min. The AVS process with these parameters leads to the formation of a functional and homogenous amine functional layer on the surface of Ti substrates, as previously shown [36].

#### 2.2.2. Covalent Immobilization of Fibronectin on Ti Substrates

The extraction of fibronectin was conducted by employing cryoprecipitated human plasma following the methodology described by Poulouin et al. [38] using gelatin-heparin chromatography affinity. This process yielded a fibronectin stock of 500 µg/mL solution in carbonate-bicarbonate buffer. In order to covalently bind fibronectin to the Ti substrates, the stock solution of fibronectin was diluted in 4-morpholine-ethanesulfonic acid (MES, Sigma-Aldrich, Saint Louis, MO, USA). AVS-functionalized samples were incubated with the fibronectin-MES solution for 1 h. Subsequently, a solution containing N-(3-dimethylaminopropyl)-N′-ethylcarbodiimide hydrochloride (EDC, Sigma-Aldrich)/N-hydroxysuccinimide (NHS, Aldrich) in MES buffer was added to the samples and incubated for 4 h. The final concentrations of the reagents were as follows: fibronectin 200 µg/mL, MES 0.1 M pH = 6.0, EDC 0.125 mg/mL, and NHS 0.0315 mg/mL.

Following the incubation period, the samples were extracted from the solution and gently rinsed with distilled water in order to eliminate any non-adhered protein from their surface. Subsequently, an intensive cleaning procedure was employed to ensure the complete removal of the surplus unreacted EDC/NHS crosslinkers. The cleaning procedure was established in a previous work [39] and consisted of incubating the samples in PBS (10 mM, pH = 7.4), MES (0.1 M, pH = 6.0), and Dulbecco’s Modified Eagle Medium (DMEM, pH = 7.4) for 5 h, 72 h, and 24 h, respectively. As indicated in [39], no sign of degradation was observed on the fibronectin during the whole preparation protocol or during subsequent cell culturing.

The different conditions assessed in this work are summarized in Table 1.

### 2.3. Stability Testing

In order to test the stability of covalently bound fibronectin on the surface of Ti substrates, the samples underwent sonication in a solution of 10% (*w*/*v*) sodium dodecyl sulfate (SDS, Fisher scientific, Waltham, MA, USA) in PBS for 1 h and were incubated in a renewed SDS solution overnight. Finally, the samples were rinsed with distilled water. The rationale behind this procedure was to disrupt any non-covalent bonds in the structure, thus solubilizing and eliminating non-covalently bound fibronectin from the surface of the samples.

### 2.4. Characterization

#### 2.4.1. Fluorescence Microscopy

Fluorescein 5(6)-isothiocyanate (FITC, Fluka, Madrid, Spain) labeling of fibronectin was performed following a method described by Hoffmann et al. [40]. Briefly, a solution of FITC in anhydrous DMSO was mixed with fibronectin solution (1:100) and the unbound FITC was removed from the solution through gel filtration.

The presence of FITC-labeled fibronectin, whether immobilized or adsorbed, was checked on the substrate surface using fluorescence microscopy (Leica DMI 3000B) at an emission wavelength of 520 nm. Images of the samples were captured both prior to and subsequent to treatment with SDS detergent to assess the stability of the bound fibronectin.

#### 2.4.2. Atomic Force Microscopy

In order to assess the presence of fibronectin, the surface of the samples was examined by atomic force microscopy (AFM, Cervantes AFM, Nanotec S.L., Madrid, Spain) before and after the treatment with SDS. The measurements were conducted in air using a pyramidal cantilever (Olympus OMCL RC800, semi-angle 39°, nominal resonance frequency 69 KHz) in dynamic mode. The obtained profile data were analyzed using WSxM 5.0 software (Nanotec S.L., Madrid, Spain) [41]. The root mean square (RMS) roughness of the samples was determined from the profile data using WSxM 5.0

### 2.5. Cell Cultures

Murine bone marrow mesenchymal stem cells (BM-MSC) were employed as the cellular model in this study. Isolation and expansion of BM cells were carried out on fibronectin-coated wells (Corning Inc., Corning, NY, USA) in Iscove’s Modified Dulbecco’s Medium (IMDM, HyClone, Madrid, Spain) supplemented with 20% of MSC stimulatory supplements (Stem Cell Technologies, Grenoble, France), 100 μmol/L, 2-mercaptoethanol (Sigma), 100 IU/mL penicillin (Sigma), 0.1 mg/mL streptomycin (GIBCO, Waltham, MA, USA), 2 mmol/L L-glutamine (GIBCO), 10 ng/mL human PDGF-BB (Peprotech, London, UK), and 10 ng/mL rm-EGF (Peprotech). Adherent cell clusters were cultured for a minimum of 5 passages. After this point, the cells were routinely maintained in DMEM (HyClone) supplemented with 10% fetal bovine serum (FBS, HyClone) and 1% penicillin/streptomycin (Sigma). All the experiments were performed using BM-MSCs in passages 5 through 15.

Prior to cell culture, the samples underwent sterilization through UV irradiation. Samples were exposed to UV light for 20 min on each side prior to cell culturing and immediately put inside the wells of a p24 multiwell. No bacteria or fungi contamination was detected in any case. BM-MSCs were seeded on all the samples at a concentration of 50,000 cells per well and incubated in a humidified atmosphere containing 5% CO_2_ at a temperature of 37 °C for either 4 or 48 h. BM-MSCs cultured on blank wells were used as controls. The experiments were performed twice using duplicate samples.

#### 2.5.1. Cell Viability

The viability of cells was assessed after 4 and 48 h of seeding by staining the cells with calcein acetoxymethyl (calcein AM, Life Technologies, Carlsbad, CA, USA, 0.5 µg/µL in DMSO) and propidium iodide (PI, Sigma-Aldrich, 750 µM in PBS). For this purpose, the samples were incubated with a solution containing 1 µL/mL calcein/AM and 1 µL/mL PI in DMEM for a duration of 30 min. Ultimately, cells were visualized by a fluorescence microscope (Leica DMIRB) at emission wavelengths of 515 and 636 nm for calcein AM and PI, respectively. Three representative images were captured for each sample using a digital camera attached to the microscope (Leica DC100) and the numbers of viable and PI-positive cells were counted in each image using ImageJ software (NIH, USA). Results were expressed as the number of viable cells per mm^2^ of the sample’s surface.

#### 2.5.2. Cell Proliferation

Cell proliferation was evaluated at 48 h after seeding on each sample using the 2,3-bis-(2-methoxy-4-nitro-5-sulfophenyl)-2H-tetrazolium-5-carboxanilide (XTT, Applichem, Darmstadt, Germany) assay according to the manufacturer’s instructions. The absorbance of each well was measured spectrophotometrically at a wavelength of 450 nm using an ELX808 microplate reader (BioTeK, Santa Clara, CA, USA). The absorbance values were then normalized by the surface area of each respective sample to account for any variations in sample size.

#### 2.5.3. Cell Spreading

At the 4 h time point after seeding, the cellular morphology was assessed. In order to do so, the cells were carefully washed with PBS and fixed using a 4% paraformaldehyde solution, followed by permeabilization using a solution of 0.1% Triton-X100 in PBS. Finally, for visualization, the cell actin filaments were stained with phalloidin tetramethylrhodamine B isothiocyanate (Phalloidin-TRITC, Sigma) and the nuclei were counterstained with hoechst 33,258 (Molecular Probes, Eugene, OR, USA) by incubating the cells in a solution containing a combination of 2 µg/mL phalloidin and 0.2 mg/mL hoechst in PBS for a duration of 60 min at room temperature.

The cells were visualized using a fluorescence microscope (Leica DMIRB) equipped with a digital camera (Leica DC100) at emission wavelengths of 570 and 461 nm for phalloidin and hoechst, respectively.

From the obtained images, the surface area and the perimeter of the cells were measured using ImageJ software. Additionally, Feret’s diameter, defined as the longest distance between any two points along a given boundary, was also determined. Fifty individual cells were measured corresponding to each group.

### 2.6. Statistical Analysis

Unless indicated otherwise, all results are based on two independent experiments, each one using duplicate samples for each group. Statistical analyses were conducted using IBM SPSS Statistics 20 software. Statistically significant differences were determined using a one-way ANOVA followed by a Games–Howell post-hoc test. A *p*-value < 0.05 was considered statistically significant. All data are presented as the mean value ± standard error.

## 3. Results

### 3.1. Evaluation of Fibronectin Attachment

#### 3.1.1. Fluorescence Microscopy

**Figure 1:**
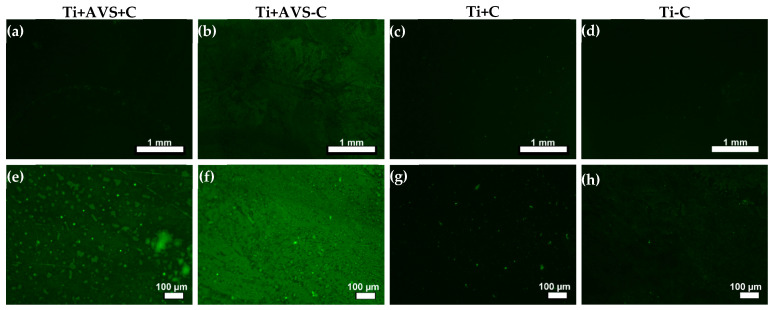
Fluorescence microscopy images of FITC-labeled fibronectin following the classification indicated in Table 1: (**a**,**e**) Ti + AVS + C, (**b**,**f**) Ti + AVS-C, (**c**,**g**) Ti + C, and (**d**,**h**) Ti − C samples in two different magnifications. Samples were incubated with an SDS solution before obtaining the micrographs.

Initially, fluorescence microscopy was employed in order to determine the presence of FITC-labeled fibronectin on the AVS-functionalized and bare Ti samples. The effect of AVS functionalization (Ti + AVS or Ti) and the presence of crosslinkers (+C or −C) on the immobilization process were evaluated. The fluorescence microscopy images are shown in Figure 1 after the samples were washed with the SDS solution. Fluorescence is significantly higher in the AVS-functionalized samples compared with the controls (non-functionalized samples). However, no clear difference is apparent from the comparison of the sample incubated with the EDC/NHS crosslinkers (+C) and that not incubated with the crosslinkers (−C). Consequently, it was necessary to use an alternative characterization technique to assess the efficiency of the crosslinking chemistry with respect to the physical adsorption of the protein on the surface.

#### 3.1.2. Atomic Force Microscopy

**Figure 2:**
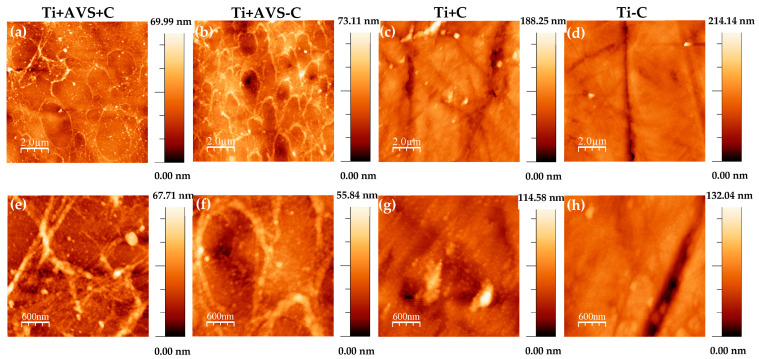
Atomic force microscopy images of (**a**,**e**) Ti + AVS + C, (**b**,**f**) Ti + AVS-C, (**c**,**g**) Ti + C, and (**d**,**h**) Ti − C samples in two different scan sizes. The images follow the classification indicated in Table 1.

**Figure 3:**
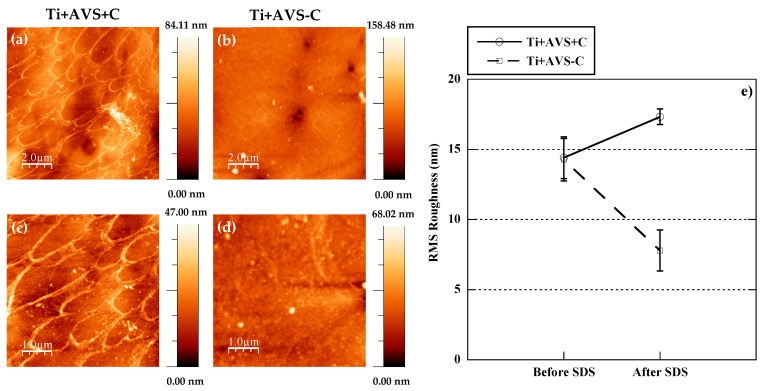
Atomic force microscopy images of (**a**,**c**) Ti + AVS + C and (**b**,**d**) Ti + AVS-C samples after treatment with SDS in two different scan sizes, and (**e**) RMS roughness of Ti + AVS + C and Ti + AVS-C samples before and after treatment with SDS. The images follow the classification indicated in Table 1.

The surfaces of all samples were characterized with AFM to assess the presence and stability of fibronectin on the substrates. AFM topography images of the samples before and after incubation with an SDS solution are depicted in Figure 2 and Figure 3, respectively. As previously observed from the fluorescence micrographs, distinct topographic characteristics indicative of the existence of fibronectin on the surface can be prominently seen in the samples Ti + AVS + C and Ti + AVS-C, in contrast with the topography observed in Ti + C and Ti − C samples.

Figure 3 illustrates the AFM topography images of Ti + AVS + C and Ti + AVS-C samples after incubation with an SDS solution. It is evident that, although fibronectin is still present on Ti + AVS + C samples after the treatment, the majority of the fibronectin on Ti + AVS-C samples is effectively removed. Figure 3e presents the RMS roughness values for Ti + AVS + C and Ti + AVS-C samples before and after the treatment with SDS. It can be seen that while the roughness for Ti + AVS + C does not change significantly after the treatment with SDS, there is a marked decrease in the roughness of Ti + AVS-C samples, indicating removal of fibronectin from these samples. Collectively, these results provide strong evidence for the enhanced stability of covalently immobilized fibronectin in comparison to the physically adsorbed protein.

### 3.2. Biological Assessment

#### 3.2.1. Cell Adhesion and Proliferation

**Figure 4:**
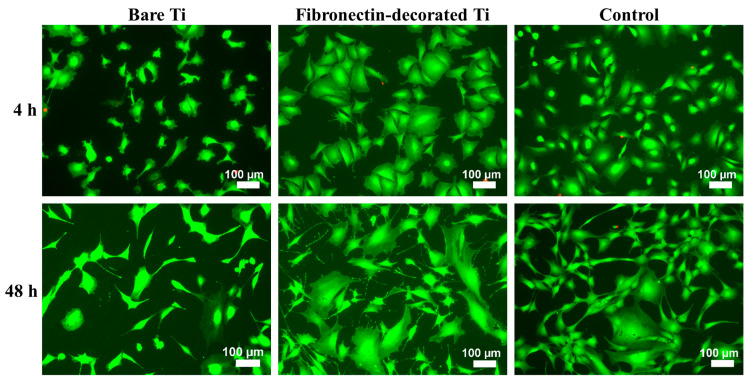
Fluorescence microscopy images of calcein/PI-stained BM-MSCs adhering to bare Ti, fibronectin-decorated Ti, and polystyrene control samples at 4 h and 48 h following seeding. Viable cells are stained green, whilst the dead cells appear as red events.

The biological response of fibronectin-immobilized (Ti + AVS + C) samples was assessed by performing in vitro cultures of murine bone marrow mesenchymal stem cells (BM-MSCs). In Figure 4, the morphology of the BM-MSCs adhered to bare Ti, fibronectin-decorated Ti, and polystyrene control samples at 4 and 48 h after seeding, is shown. At 4 h after seeding, the cells adhering to fibronectin-immobilized samples exhibited a more pronounced extension on the surface in comparison to those on bare Ti. After 48 h of seeding, the cells on fibronectin-decorated Ti displayed a superior cell arrangement, more developed cellular processes, and extended filopodia compared to those observed on bare Ti. As also observed in Figure 4, there is very reduced number of dead cells compared with the number of viable cells. Although the cause of cell death cannot be determined from these studies, these results preclude the existence of significant apoptosis of the cells resulting from their interaction with the material. This fact is consistent with the high biocompatibility conventionally assigned to titanium implants. No statistically significant differences were found between the different samples characterized within each group.

**Figure 5:**
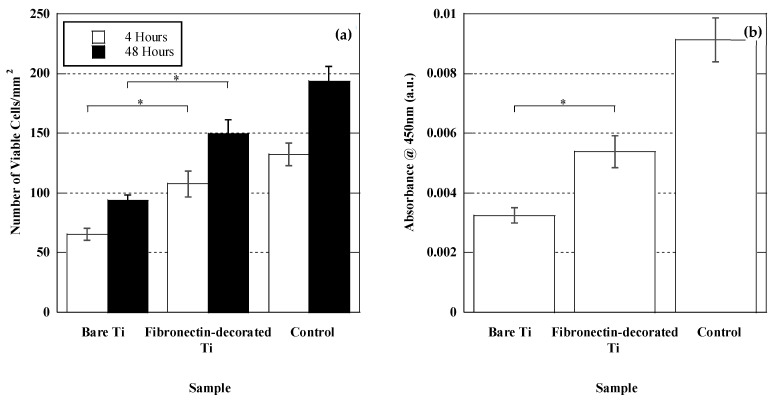
Number of BM-MSCs on bare Ti, fibronectin-decorated Ti, and polystyrene control samples obtained by (**a**) cell counting from micrographs after 4 and 48 h of seeding and (**b**) XTT measurement 48 h after seeding. * depicts statistically significant difference.

Figure 5a depicts the number of calcein-positive cells counted on each sample. The results demonstrated a significantly higher number of adhered cells on the fibronectin-decorated Ti samples compared to the bare Ti samples, both at 4 and 48 h after seeding. (*p*-value = 0.009 and 0.002 for 4 and 48 h, respectively).

In addition to the cell quantification obtained from the analysis of calcein/PI-stained samples, independent XTT assays were conducted after 48 h of seeding (Figure 5b), since the XTT assay is used to measure cellular metabolic activity as an indicator of cell viability, proliferation, and cytotoxicity. The XTT assays confirmed the results obtained from the calcein/PI experiments and showed that the fibronectin-decorated Ti samples exhibited a significantly higher number of metabolically active cells in comparison to the bare Ti (*p*-value = 0.013).

#### 3.2.2. Cell Morphology and Spreading

**Figure 6:**
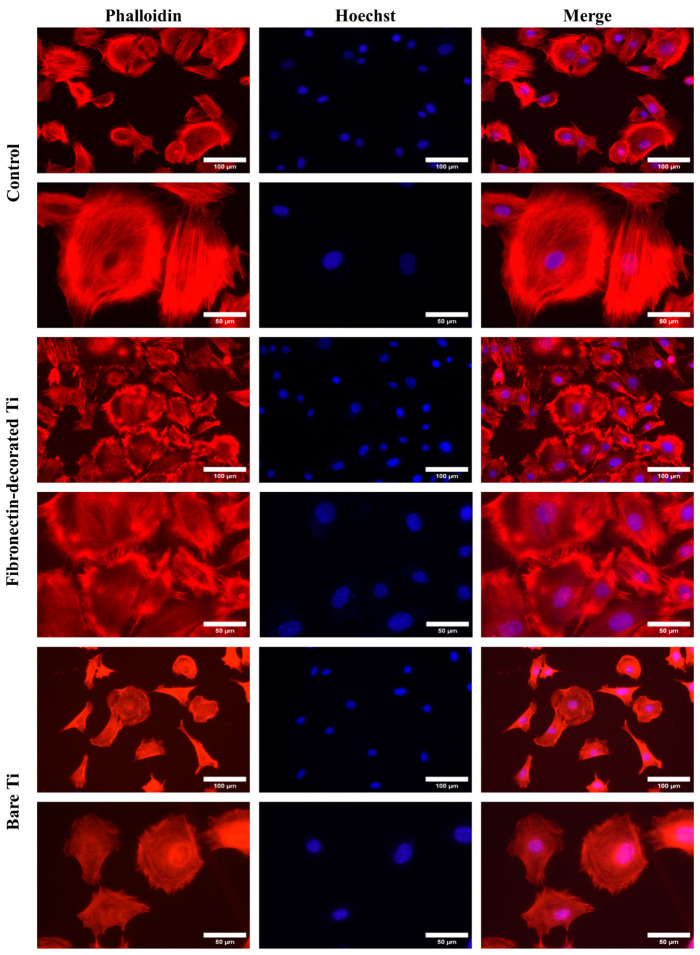
Fluorescence microscopy images of phalloidin/hoechst-stained BM-MSCs adhering to bare Ti, fibronectin-decorated Ti, and polystyrene control samples 4 h after seeding at two different magnifications.

In Figure 6, the fluorescence images of phalloidin/hoechst-stained BM-MSC cells adhering on bare Ti, fibronectin-decorated Ti, and polystyrene control samples at 4 h after seeding are shown. The cells adhering on fibronectin-decorated Ti samples exhibited a more mature and developed actin cytoskeleton in comparison to those adhering on bare Ti samples.

**Figure 7:**
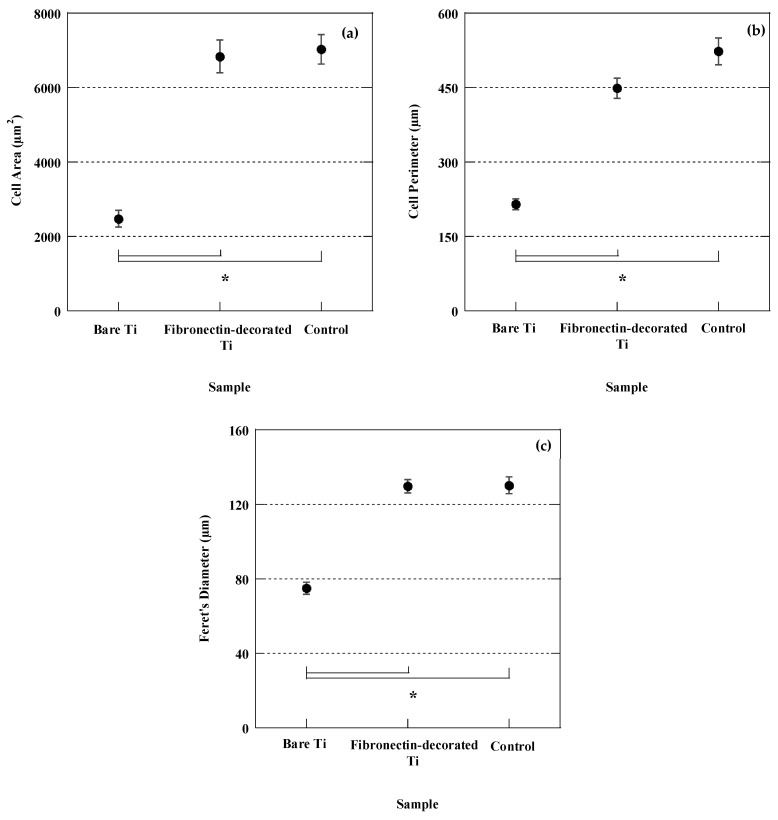
Cell surface area (**a**), cell perimeter (**b**), and cell Feret’s diameter (**c**) of BM-MSCs adhering to bare Ti, fibronectin-decorated Ti, and polystyrene control samples at 4 h after seeding. * indicates statistically significant difference.

Quantitative measurements of cell surface area as shown in Figure 7a revealed that the cells adhering to bare Ti samples possess the smallest surface area. The cells adhering on the fibronectin-decorated Ti samples possessed a significantly larger surface area, almost three times larger than that of the cells on bare Ti (*p*-value = 0.0001), a value comparable to that found in the polystyrene control samples. A similar trend was observed with respect to the cell perimeter and Feret’s diameter between cells cultured on the fibronectin-decorated samples and those on control bare Ti.

## 4. Discussion

In the present study, a robust biofunctionalization procedure was developed to covalently bind fibronectin to the surface of a solid biomaterial. The procedure begins with the deposition of a thin functionalized layer on the surface of the Ti substrate using AVS [37,42]. The stability of the functionalized layer had been assessed in a previous work using nanoscratch tests [43]. It was found that the critical failure load of the amine layer deposited on Ti substrates was roughly 70 mN (a value which is approximately 25% of that of a typical hard coating, such as tungsten carbide on steel), and the mechanism of failure was determined to be the plastic deformation of the deposited layer, which led to its cohesive failure. In particular, no decohesion between the functionalized thin film and the substrate was detected during the nanoscratch tests [43].

**Figure 8:**
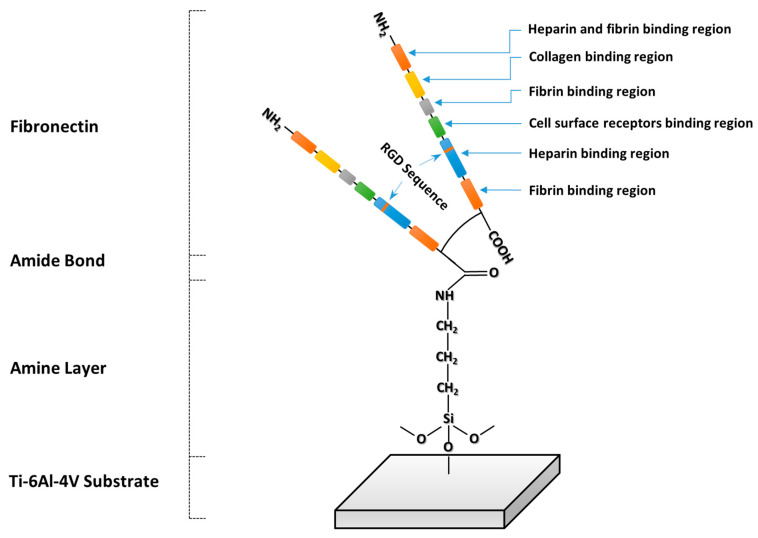
Scheme of the covalent binding of fibronectin to the functionalized titanium substrate, depicting the main cell binding motifs.

The amines present on the surface of the thin layer were used to covalently bind fibronectin with the EDC/NHS crosslinking chemistry, as shown schematically in in Figure 8.

The presence of fibronectin on the surface of the functionalized samples was initially established with fluorescence microscopy and subsequently confirmed through AFM micrographs. In order to verify the attachment of fibronectin to Ti + AVS + C samples through covalent immobilization, a process of elution with SDS was applied, since the detergent SDS is commonly used to eliminate weak interactions between proteins. The AFM topography images recorded prior to the treatment with SDS demonstrated the formation of an interconnected network structure consistent with the presence of fibronectin on Ti + AVS + C and Ti + AVS-C samples. The assembly of fibronectin interconnected network structures observed in this work is similar to those previously reported by Rico et al. [44,45].

The AFM topography images recorded after the treatment with SDS clearly confirmed that fibronectin was only present on Ti + AVS + C samples, whereas it was removed from the surface of Ti + AVS-C samples (i.e., not incubated with the EDC/NHS crosslinkers). The presence of a significant amount of fibronectin on the Ti + AVS-C before being immersed in an SDS solution can be explained by considering the potential electrostatic interaction between the functionalized substrate and fibronectin. The isoelectric point of fibronectin has been reported to occur at pI = 5.2 [46]. In this case, considering that the experiments were conducted at pH 6.0, it may be assumed that fibronectin will be negatively charged, while the surface of AVS-functionalized samples carries a positive charge due to the presence of primary/secondary amines. Consequently, the electrostatic attraction between the surface amines and fibronectin may lead to the adsorption of relatively high amounts of fibronectin on the surface of the samples. In contrast, non-functionalized samples (Ti + C and Ti − C) present a TiO_2_ layer on the surface [47] and, since this oxide layer is negatively charged at pH = 6.0 [48,49], the repulsive forces between the surface of the Ti + C and Ti − C samples and fibronectin must largely prevent the physical adsorption of fibronectin to the surface of the material.

The response of BM-MSCs to the covalently bound fibronectin on Ti samples was also addressed. It was demonstrated that a significantly higher cell number was measured on the fibronectin-decorated Ti compared with bare Ti samples at 4 h after seeding. This trend was also found in the analysis performed at 48 h after seeding.

It has been reported that the interaction of fibronectin with cells involves mechanisms that rely on the recognition of cell adhesion motifs by integrins [50]. These motifs, such as RGD [51,52,53], PHSRN (Proline—Histidine—Serine—Arginine—Asparagine) [19,54], LDV (Leucine—Aspartic acid—Valine) [21], and REDV (Arginine—Glutamate—Aspartic acid—Valine) [20], are present in the sequence of fibronectin. Consequently, it may be hypothesized that some or all of these motifs may contribute to the enhanced proliferation of BM-MSCs on the fibronectin-decorated substrates.

Moreover, an enhanced adhesion of cells to the substrate can intricately modulate the interaction between the cells and the material. It is widely acknowledged that the proliferation of substrate-dependent cells is profoundly influenced by the extent of cellular spreading on the material. Notably, insufficient spreading on a substrate has been identified as a potential trigger for cellular apoptosis [55]. Alternatively, it is also hypothesized that the magnitude of initial cell spreading may positively influence subsequent cell proliferation [56,57,58], owing to two potential mechanisms at play. The first mechanism suggests that cells with a larger surface area have an increased capacity to uptake a greater quantity of proteins from the surrounding biological fluids. This elevated protein uptake promotes the transition of cells from G0 to G1 and from G1 to S phase of the cell cycle, consequently favoring cell proliferation [59,60,61,62]. The second mechanism involves mechanical cues, as detected by the actin cytoskeleton. As cells undergo spreading and expansion on the substrate, the stress exerted on the actin fibers increases. This increased stress within the actin fibers subsequently stimulates cell proliferation through DNA synthesis and nuclear expansion [63,64,65,66]. Therefore, an enhanced initial cell spreading by virtue of covalent binding of fibronectin to an AVS-functionalized implant could improve subsequent cell proliferation and, possibly, the integration of the material in the recipient organism due to the possible enlarged tissue–implant contact area. Elongated cells with a large Feret’s diameter, as observed on the fibronectin-decorated Ti samples, suggest a highly expanded cytoskeleton with stretched actin fibers, which is favorable for cell proliferation.

## 5. Conclusions

In this study, fibronectin was covalently bound to a functionalized Ti substrate through the EDC/NHS crosslinking chemistry. The presence of the fibronectin on the surface was characterized by fluorescence microscopy and by AFM. It is demonstrated that higher levels of fibronectin were present on functionalized samples compared with bare Ti (control) samples. The robustness of the procedure was confirmed by the resistance of the immobilized fibronectin to incubation in SDS. In vitro cell cultures revealed that the covalent binding of fibronectin on the Ti surface improves initial cell adhesion and spreading of BM-MCSs compared with bare Ti samples, reaching values comparable to those of the polystyrene controls. In summary, the results discussed in this work represent a robust approach for the covalent immobilization of fibronectin in order to enhance the biological response of a wide range of biomaterials. Although more in vitro and preclinical studies will be required before validating this approach for the production of novel prostheses with the ability to modulate the reaction of the organism to the implant, the versatility and robustness of this procedure clearly offers a promising pathway for their development.

## Figures and Tables

**Table 1:** Terminology used for the identification of different samples.

Sample	Details
Ti + AVS + C	AVS-functionalized Ti-6Al-4V incubated with fibronectin and EDC/NHS crosslinkers
Ti + AVS-C	AVS-functionalized Ti-6Al-4V incubated with fibronectin solution without addition of EDC/NHS crosslinkers
Ti + C	Bare Ti-6Al-4V incubated with fibronectin solution and EDC/NHS crosslinkers
Ti−C	Bare Ti-6Al-4V incubated with fibronectin solution without addition of EDC/NHS crosslinkers

## Data Availability

Data are available upon request to the corresponding author.
